# Affective Neuroscience Personality Scale (ANPS) in Children with Internalizing Disorders

**DOI:** 10.3390/pediatric17030055

**Published:** 2025-05-08

**Authors:** Simone Cupellaro, Valentina Colonnello, Ugo Sabatello, Chiara Ubertini, Carla Sogos

**Affiliations:** 1 Department of Human Neuroscience, Sapienza University, Via dei Sabelli 108, 00185 Rome, Italy; simone.cup81@yahoo.it (S.C.); ugo.sabatello@uniroma1.it (U.S.); 2 Department of Medical and Surgical Sciences, University of Bologna, Via Massarenti 9, 40138 Bologna, Italy; valentina.colonnello@unibo.it; 3 ASL RM 1, Local Sanitary Authority, Rome 1, Via Boccea, 271, 00167 Rome, Italy; chiara.ubertini@aslroma1.it

**Keywords:** ANPS, affective neuroscience, internalizing disorders, children

## Abstract

**Background/Objectives**: This study of endophenotypes represents a new research approach to overcome the limits of a syndromic model to psychiatric diseases. The Affective Neuroscience Personality Scales (ANPS, 31) is a self-report questionnaire used to facilitate the transition from the syndromic to the endophenotypic model through the assessment of basic emotional systems described by Panksepp (1): SEEKING, PLAY, CARE, FEAR, RAGE, PANIC, and LUST. The ANPS was used with adults, but it may be important to investigate the expression of basic emotional systems in childhood clinical disorders. **Methods**: The present study compares the ANPS scores of a group of children (*n* = 71) with internalizing disorders (diagnoses of depression and anxiety) with those of a normative group (*n* = 208) (8–13 years). **Conclusions**: We found that the group with internalizing disorders showed significantly lower scores for SEEKING and PLAY and significantly higher scores for ANGER and SADNESS than the control group. Furthermore, depressed children reported significantly lower scores in the SEEKING, PLAY, CARE scales and higher scores in the ANGER and SADNESS scales than healthy children. The children with anxiety disorder had significantly lower scores in the SEEKING and PLAY scales and higher scores in the FEAR and SADNESS scales than control children. No significant effect was found in reference to the age of the children. The results indicate that the ANPS might be a useful instrument to assess the expression of emotional endophenotypes in childhood.

## 1. Introduction

Affective neuroscience [1] has played a key role in identifying a mind–body link and promoting interaction between clinical and laboratory experience in order to better understand the processes underlying motivation and social behavior. As demonstrated by Jaak Panksepp, the founder of cross-species affective neuroscience, every behavior, decision, or action is influenced by “primary emotional systems” [1,2]. The identification of emotional systems is based on the recognition of the specificity of the neurobiological reality of emotions. As demonstrated by studies on animal models, each system, if activated through electrical stimulation, induces a specific distinct emotion [1,3]. The seven emotional systems identified are (1) SEEKING, the enthusiasm that stimulates curiosity and facilitates discovery of the world by overcoming a state of inertia; (2) FEAR, which is based on avoidance of pain and danger in order to promote survival through flight or freezing; (3) ANGER, which allows individuals to attack and fight to free themselves from potentially dangerous situations; (4) PANIC/Sadness, which evolves from the same system as the perception of physical pain and mediates the formation of attachment bonds; (5) nurturance CARE, e.g., taking care of offspring; (6) LUST, which promotes the reproductive activities that support the species; and (7) PLAY, the system involved in the positive emotional experience that typically accompanies play [1].

One of Panksepp’s most significant contributions was his emphasis that emotions are not generated at the neo-cortical level. A decorticated animal is able to express all the emotions mentioned above. Each emotional system is associated with specific subcortical circuits and with different neuromodulators [1,4]. These systems also differ from the more primitive and phylogenetically archaic feelings with a sensory value (e.g., pain and taste) or homeostatic function (e.g., hunger and thirst). The ability to experience and express emotions is independent of the ability to recognize them. Panksepp [1] highlighted that basic emotional systems are all subcortical and are shared by humans and other mammals. 

The SEEKING system begins from the ventral tegmental area (VTA) towards the medial forebrain fasciculus and the lateral hypothalamus, the nucleus accumbens, and the medial prefrontal cortex through the mesolimbic and mesocortical dopaminergic pathways [3]. In humans, the system reaches the sensory-perceptive cortices collected in the posterior part of the brain [3]. With reference to the neurochemical factors involved, the SEEKING system is mainly fueled by the neurotransmitter dopamine, but other neuropeptides such as glutamate, orexin, and neurotensin also play a significant role [5,6].

Siegel [7] has shown that the ANGER system extends from the medial areas of the amygdala, down the semicircular pathway of the stria terminalis, to the medial hypothalamus and the areas of the periaqueductal gray (PAG). Electrical stimulation of these areas in humans produces intense feelings of anger, a tendency to clench the jaw, and a strong increase in blood circulation in the PAG area [8]. The main substances that activate the ANGER system are testosterone, substance P, and norepinephrine, while glutamate, acetylcholine, and nitric oxide are nonspecific brain activators [7]. The ANGER system is inhibited by endorphins and endogenous opioids, oxytocin, and serotonin.

The main pathway of the FEAR system comprises between the basolateral and central regions of the amygdala (and other higher brain areas, including the lithotripsy nucleus of the striatum terminalis and the lateral septal area), down through the anterior and medial hypothalamus to the periaqueductal gray (PAG) of the midbrain and the adjacent tegmental field [1,9,10,11,12]. Electrical stimulation of the FEAR system can generate a variety of responses in animals even when there are no fearful stimuli, such as wariness, frozen immobility, and escape behaviors [3]. Humans who are stimulated in these brain areas report fear, anxiety, and a sense of oppression [13]. The FEAR system can be activated by the corticotropin-releasing factor (CRF), neuropeptide Y (NPY), cholecystokinin (CCK), catecholamines, neuropeptide alpha-MSH, glutamate, and adrenocorticotropic hormone (ACTH) [3].

The CARE system is linked to the activation of several chemicals, including brain dopamine, opioids, and oxytocin [14,15]. Oxytocin is one of the main chemicals in the maternal condition and is produced in the female brain in higher amounts than in the male brain [16]. Estrogens regulate the production of oxytocin through the cell fields of the anterior hypothalamus, including the paraventricular nucleus (PVN) and the dorsal preoptic area (dPOA). The CARE circuit extends through the hypothalamus to the ventral tegmental area (VTA) [17].

The anatomical basis of the PANIC emotional system, which mediates separation anxiety vocalizations and social feelings of loss, involves the periaqueductal gray (PAG), surrounding midbrain regions, dorsomedial thalamus, ventral septal area, dorsal preoptic area, and sites in the bed nucleus of the stria terminalis [18]. In humans, strong electrical stimulation of PANIC brain regions can induce a state of despair that ends when the stimulation is stopped [19]. Brain neuropeptides that reduce the unpleasant sensations of the PANIC system are the endogenous opioids, oxytocin, and prolactin.

Subcortical areas involved in the PLAY system include midline thalamic regions, such as the parafascicular complex and the posterior dorsomedial nucleus [3]. Nonspecific neurochemicals implicated in the PLAY system (specific neurochemicals have not yet been identified) include opioids, of which low doses in animals promote playfulness and social dominance, while high doses reduce social and playful behaviors [20]; meanwhile, oxycytidine and corticotropin-releasing hormone have been shown to reduce play [21].

In male mammals, the center of the LUST system is located in the medial regions of the anterior hypothalamus, with differences in some species, since, for example, in rats, it corresponds to the preoptic area (POA), while in humans, it corresponds to the interstitial nuclei of the anterior hypothalamus (INAH) [3]. The organization of sexual circuits begins during fetal life, leading to the development of differences that are controlled by testosterone. In adolescence, females enter puberty due to the action of estrogens and progesterone secreted by the ovaries, while males develop puberty through testosterone produced by the testes [3].

In the clinical field, affective neurosciences emphasize a transition from the classic nosology of psychiatric syndromes based on the description and classification of symptoms (i.e., a categorical approach) to an expansion of the clinical picture through the observation of the manifestations of “emotional endophenotypes” [22,23]. “Endophenotypes” represent an approach that focuses on “measurable components invisible to the naked eye along the pathway between disorder and genotype” [24]. These “components” involve biological, genetic, cognitive, and psychological markers; for example, the psychological trait of temperamental negative affectivity has been associated with hypervigilant cognitive attention to threatening stimuli, hyperactivity of the amygdala, and a short allele of the serotonin transporter gene [25,26]. The identification of endophenotypes could contribute to broadening the knowledge of mental disorders, as they can improve the understanding of the mechanisms underlying mental disorders, reducing the gap between genes and behavior through the study of neural and neurochemical circuits [27,28,29]. This model, based on neuro-ethological and human neuroscientific research, assumes that socio-affective skills are linked to the neurodynamics of basic brain emotional systems and that their excessive, reduced, or unbalanced activity may be involved in the formation of psychiatric disorders [23,30,31].

Attention to basic emotional aspects also allows us to take into account the quality of subjective experience. This approach can be useful with children, who tend to express their discomfort in non-verbal ways (withdrawing, becoming agitated, worrying, attacking objects, etc.), which in turn can be linked to certain emotional states. The ability to experience and manifest emotions, in fact, is independent of the ability to recognize them. The most primitive form of consciousness, “affective consciousness”, is experiential, based on the experience and expression of basic emotions. The forms of “secondary” consciousness reflect the ability to elaborate thoughts on lived experiences and to understand how external events are associated with internal ones. Panksepp [4] identified some tertiary forms of consciousness, linked to the activation of neocortical areas that develop over time with social interaction and are based on the metacognitive ability to formulate reflections on one’s own thoughts, to represent oneself and emotional states as objects of reflection [4]. Primary and secondary forms of consciousness are also found in lower animals; tertiary forms are characteristic of humans and are linked to the maturation of neocortical areas that allow linguistic–symbolic transformations of thoughts and the ability to mentalize. The recognition of three levels of consciousness is useful for understanding that psychological distress, especially in children, can be experienced independently of the ability to represent and describe it through linguistic and/or symbolic tools. A child, for example, can experience a feeling of deep sadness or fear on an emotional and somatic level without being able to describe and reflect on this experience. It may therefore be important to investigate the expression of subcortical emotional systems in children since attention to basic emotional aspects allows us to take into consideration the quality of subjective experience.

Internalizing disorders, such as depression and anxiety, are among the most frequent forms of psychopathology in the pediatric age [32,33] that interfere with the ability to engage in developmentally appropriate tasks and activities. In the field of affective neuroscience, the onset of depression is thought to result from a change in the balance between basic emotional systems since depression is associated with decreased activation of the SEEKING system in favor of hyperactivation of the PANIC system [2,34,35]. The role of SEEKING in depressive disorders has also been demonstrated in human research. The electrical stimulation of the SEEKING circuit can lead to antidepressive effects in individuals with depressive forms resistant to pharmacological treatment [2]. At the behavioral level, animal models have demonstrated that separation from the reference figure induces protest and SEEKING responses from the child that can lead to despair if this search does not lead to any result and if the vocalizations of PANIC/distress (equivalent to infant cries) are not followed by parental care [36,37]. Panksepp [35] identified the core of depressive feelings in the pain linked to the loss of emotionally significant figures and in withdrawal and divestment from the outside world as a natural defense mechanism to conserve the few resources necessary for survival. Some studies [38,39] have found that the proportion of loss events in childhood, such as death or separation from parents, was significantly higher in depressed children than in control groups. The pain of loss can also be linked to events that have a strictly personal and symbolic meaning, for example, the emotional distancing of the caregiver or partner [40].

With regard to anxiety disorders, neuroscience has deepened the link between anxiety disorders and the neurobiological circuits of fear since fear constitutes an emotional state that is strongly activated in anxious symptoms. Affective neuroscience has shown that mammalian brains contain the unconditional circuits of the FEAR system, which are concentrated in subcortical brain regions [1]. Animals show evident fear responses during electrical and chemical stimulation of specific brain regions [12,41,42], and humans who are stimulated in the same subcortical areas report experiences of fear and anxiety [43,44,45]. This implies that the neural substrates of anxiety and fear can be analyzed with neuroscience tools supported by ethological analysis of animal behavior [46]. Panksepp [1,47] argued that the main aspect of the subjective component of fear is related to the specific subcortical system of the FEAR system. This system also coordinates behavioral responses to potential threats, causing animals to move, hide, or freeze based on the threat. The clinical symptoms of most anxiety disorders appear to overlap with FEAR system activation since they are characterized by excessive worry combined with feelings of anguish. Generalized anxiety disorder is characterized by a variety of symptomatic manifestations, such as uncontrollable apprehension, excessive alertness, nervousness, and restlessness. Panksepp [4] suggested that many anxiety disorders may result from changes in the sensitivity of the subcortical system of the FEAR system. The subcortical components of the FEAR system can become hypersensitive in response to chronic hyperactivation [48,49].

A diagnostic formulation that takes these aspects into consideration can help identify the emotional core of distress. Identifying the balance of the basic emotional systems is essential in order to determine clinical characteristics in the diagnostic phase and the most effective psychotherapeutic path.

To facilitate the transition from the syndromic to the endophenotype model, Davis, Panksepp, and Normansell [31] developed the Affective Neuroscience Personality Scales (ANPS) to identify the prevalence of dominant affects and, therefore, the activation of corresponding subcortical systems in normative and clinical populations. Over the years, the ANPS has been translated from English to several languages [50] and has demonstrated good psychometric properties in normative samples, as well as construct validity and internal reliability [51,52]. ANPS has also been tested in the clinical field [53,54,55,56,57], in which a significant relationship was observed between the alteration of some neuroemotional systems and specific psychiatric disorders, which promoted stimulating reflections in clinical activity [50,58].

Although the ANPS was built and used for adults, it would be interesting to investigate the expression and dynamic balance of basic emotional systems in different stages of development and in clinical disorders in childhood since the manifestation of basic systems is also evident in children [59]. Identifying the basic emotional aspects in pediatric depressive and anxiety disorders would allow a better understanding of the subjective experience. Children can express their discomfort in various forms (e.g., closing up, fidgeting, worrying, divesting on school and peers), which in turn can be linked to different affective patterns [60]. Recently, the ANPS has been adapted for a normotypic population of children [61]. To make the tool more relevant to the developmental age, a brief version of the ANPS with 50 items was built, eliminating statements that are not understandable or unsuitable for children or modifying unclear sentences. The scales relating to the six neuroemotional systems (SEEKING, PLAY, CARE, ANGER, FEAR, and SADNESS) were maintained, while the scale relating to Spirituality was not included, whose contents were deemed not relevant for the pediatric age. In this study [61], the distribution of the neuroemotional systems with respect to the age/gender variables and the concurrent validity of the ANPS with the BIG-FIVE Children Questionnaire [62] was explored on a normative sample of school age (from 8 to 13 years). The results were consistent with previous research on adults [31,63,64,65,66] and showed moderate construct and concurrent validity of the instrument. In particular, no significant effect was found in reference to the age of the children, while a significant effect of the variable gender emerged, in which females scored higher than males on the CARE scale. Second-order factor analysis highlighted two main components: negative global affect and positive global affect. As in Davis’s study [31] and in the subsequent Italian validations [67,68], the first component is given by PANIC, FEAR and ANGER, while the second component, positive global affect, is given by the common factors CARE, SEEKING, and PLAY. 

Since no study has investigated the affective neuroscience model in childhood clinical disorders, the purpose of this work was to apply the ANPS to pediatric psychopathological conditions. This research was conducted on a group of children with internalizing disorders, in particular with a diagnosis of depression or anxiety, compared with a control group composed of children with normotypical development in order to identify possible differences in primary emotional systems [69] in terms of ANPS scale activation. In sum, we hypothesized (a) a reduced influence of the age and sex variables in the distribution of the values of the neuroemotional systems, (b) a significant correlation between the scales relating to “positive affect” (SEEKING, PLAY, CARE) and a significant correlation between the scales relating to “negative affect” (FEAR, ANGER, SADNESS), and that (c) children in the clinical group, compared with the control group, would show lower scores in the so-called “positive” affective dimensions and higher scores in the “negative” ones.

## 2. Materials and Methods

### 2.1. Participants

The total sample comprised 279 children divided into two main groups. The first group (control) was composed of 208 children with normotypic development and average social background aged 8 to 13 years (mean age: 10 years, standard deviation (SD): 1.7), of which 127 were males and 81 were females. Subjects reported by teachers for cognitive or physical deficits or with psychopathological problems were excluded. The second group (clinical) was composed of 71 children aged 8 to 13 years (mean age: 9.3 ± 1.5 years) of average social background, of which 43 were males and 28 were females, from the developmental psychopathology service of the university department with a diagnosis of depressive disorder (*n* = 44) or anxiety disorder (*n* = 27). Subjects with cognitive or physical deficits were excluded.

### 2.2. Instruments

ANPS: A shortened 50-item version of the ANPS self-report questionnaire was used, linguistically adapted, and validated for children [61]. This version was developed by removing items that were difficult to understand or unsuitable for children, clarifying ambiguous statements from the original ANPS [31], and excluding the spirituality subscale, which was deemed inappropriate for children. Previous validation [61] identified a two-factor structure (positive vs. negative emotions). The adapted ANPS measured SEEKING (Cronbach’s α = 0.512), FEAR (Cronbach’s α = 0.70), RAGE (Cronbach’s α = 0.48), CARE (Cronbach’s α = 0.59), PLAY (Cronbach’s α = 0.67), and PANIC (Cronbach’s α = 0.67).

K-SADS-PL DSM-5: It is a diagnostic interview [70] for the evaluation of psychopathological disorders in children and adolescents according to DSM-5 criteria. This tool provides an overall score that takes into account data collected from various sources (parents, children, teachers, and pediatricians). It consists of an unstructured introductory part, a diagnostic screening interview, a checklist for the administration of diagnostic supplements, five diagnostic supplements (mood disorders, psychotic disorders, anxiety disorders, attention deficit disorders and disruptive behavior, and substance abuse) for each of which the criteria required by the DSM-5 are provided, and an overall checklist of the patient’s clinical history. We used the K-SADS DSM-5 because, through the clinical interview, it allows for making a psychopathological diagnosis, taking into consideration the information provided by both the parents and the children.

### 2.3. Procedure

After contacting school principals for permission to conduct this study, the purpose and modalities of the procedure were explained to the parents of the children. Subsequently, children and parents who gave their consent to be part of the research were informed in clear language of the reason for the study, namely, in order to investigate how children feel emotions, react, and behave in certain situations. Children in the control group took part in the study during school hours. For children in the clinical group, the administration procedure was carried out as part of specialist visits to the psychopathology service of the university department. All parents provided informed consent, and the purposes of the study were explained to the children in a simple way. In particular, the interview was conducted by an expert clinician, who met the parents together with the child and collected information from both. During the meeting, there was a break to allow the child to rest and then resume the activity.

### 2.4. Statistical Analysis

Data were analyzed using the Statistical Package for Social Sciences (SPSS) version 23.0 statistical analysis program. To investigate the effect of gender and age on the individual score size of the ANPS, a repeated measures ANOVA was performed in which the total scores of the individual scales were considered as a within-subjects factor, the gender factor as a between-subjects factor, and the age factor as a covariate. The analysis of variance (ANOVA) procedure for repeated measures was performed with the group variables (clinical vs. control) treated as between-subject factors and the subscales (SEEKING, FEAR, CARE, ANGER, PLAY, and SADNESS) as within-subject factors in order to examine the difference between subjects in the control group and the clinical group.

## 3. Results

### 3.1. Distribution of Neuroemotional Systems for the Age and Sex Variables

In reference to the distribution of neuroemotional systems for the age and sex variables, no significant effect was found in reference to the age of the children for *p* > 0.05 [F (1, 275) = 17.5 *p* 0.93, *p* Hη2 0.00], while there was a significant effect of the sex variable for *p* > 0.05 [F (5, 1375) = 5.16 *p* 0.001, *p* Hη2 0.02], in which females from both the control and clinical groups have higher scores than males on the CARE scale. Furthermore, in the clinical group, males have higher ANGER values than females.

### 3.2. Intercorrelations Between the ANPS Scales in the Control and Clinical Groups

The analysis of the intercorrelations between the ANPS scales in the control (Table 1) and clinical (Table 2) groups confirmed that the SEEKING, PLAY, and CARE scales (concerning positive affect) are correlated. The FEAR, ANGER, and SADNESS scales (included in the negative affect) are also significantly correlated with each other. With respect to the primary analysis comparing the control and clinical groups across the six ANPS subscales, a post hoc sample size calculation using G*Power software [71] indicated that at least 28 participants were needed to achieve a statistical power of 0.95 with an alpha level of 0.05, assuming a medium effect size and a correlation of 0.50 between repeated measures. 

### 3.3. Comparison Between the Control Group and the Clinical Group with Internalizing Disorders

**Figure 1:**
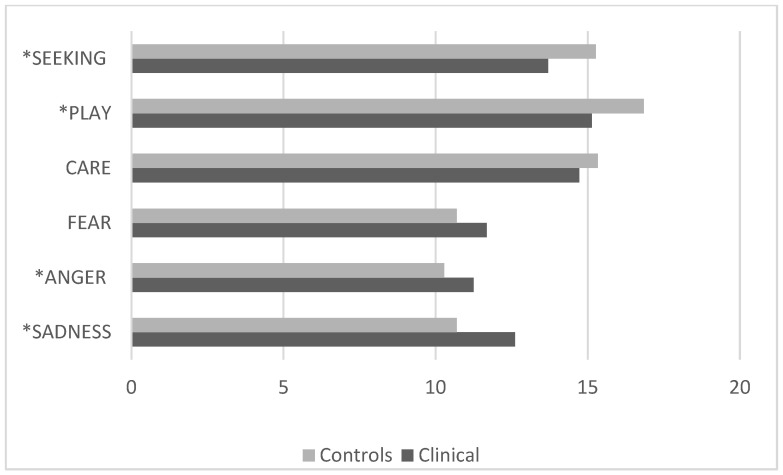
Children with internalizing clinical disorders vs. controls (ANPS scale average scores). * ANPS scales with statistically significant differences.

The comparison between the group of children with normotypic development and the clinical group with internalizing disorders revealed statistically significant differences for *p* < 0.05 [F (5, 335) = 17.5 *p* < 0.001, *p* Hη2 0.21]. In particular, the clinical group showed significantly lower scores for SEEKING and PLAY and significantly higher scores for ANGER and SADNESS than the control group, regardless of sex (Figure 1).

### 3.4. Comparison Between the Control Group and the Clinical Group with Depressive Disorders

**Figure 2:**
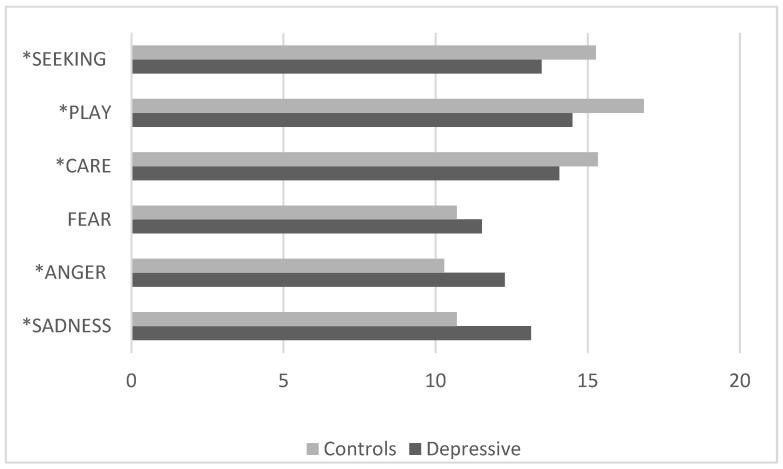
Children with depressive disorders vs. controls (ANPS scale average scores). * ANPS scales with statistically significant differences.

The comparison between the clinical group with depressive disorders and the control group (Figure 2) revealed that children with depressive disorders had significantly lower values [F (15, 1220) = 3.01 *p* < 0.001, *p* Hη2 0.03] on the SEEKING, CARE, and PLAY scales and significantly higher scores on the ANGER and SADNESS scales.

### 3.5. Comparison Between the Control Group and the Clinical Group with Anxiety Disorders

**Figure 3:**
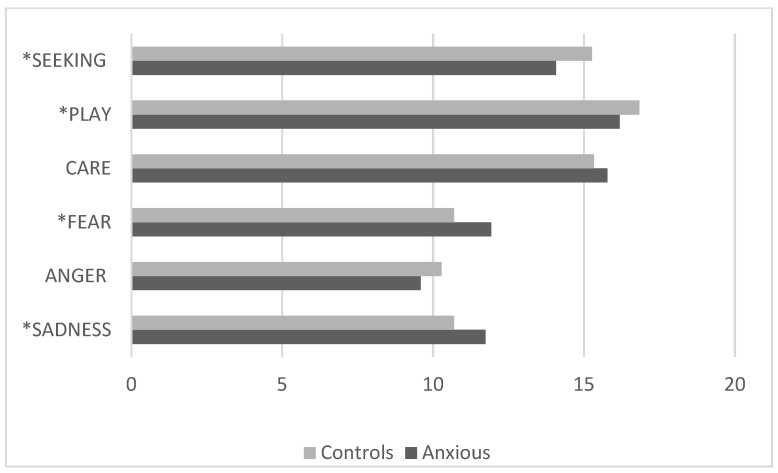
Children with anxiety disorders vs. controls (ANPS scale average scores). * ANPS scales with statistically significant differences.

The comparison between the clinical group with anxiety disorders and the control group (Figure 3) showed that children in the clinical group had significantly lower scores [F (5, 389) = 7.3 *p* < 0.0001, *p* Hη2 1.0] on the SEEKING and PLAY scales and higher scores on the FEAR and SADNESS scales.

## 4. Discussion

In the research presented, there were no significant differences in the subjects’ scores in relation to age and gender, except for the significant correlation between male gender and the ANGER dimension in the clinical group and female gender and the CARE dimension in both groups (control and clinical). The latter result was also found in the adult population. In particular, in studies by Davis et al. [31] and Pahlavan et al. [63] on normotypic subjects, men had higher scores than women on the SEEKING and PLAY scale, while women had higher scores on CARE and SADNESS. Pingault et al. [65] found higher scores for men in the PLAY system and higher scores for women in the CARE, SADNESS, and FEAR dimensions. These results are in line with the body of research showing that females show a greater propensity for caring and empathizing with others than males [72,73]. The higher values in ANGER by the males from the clinical group could be explained by the data reported in the literature, in which it is reported that, in the developmental age, anger is related to depression especially in children and adolescents of male sex. Several researchers have suggested that depression in boys manifests itself in externalizing behaviors [74,75,76,77]. In fact, research shows that boys are more likely to express their discomforts through behavioral problems than girls [78], that the worldwide prevalence of conduct disorder is three times greater in males than in females [79], and that male adolescents with depression can be characterized by the presence of externalizing aggressive behaviors [75]. These findings suggest that depression in male children may be related to anger. Furthermore, the research data that showed the absence of a difference with respect to age indicates a continuity and stability of the expression of neuroemotional systems during development. The intercorrelations found between the SEEKING, CARE, and PLAY scales, on the one hand, and the ANGER, SADNESS, and FEAR scales, on the other hand, both in the control group and in the clinical one corroborate the idea that positive and negative affect could be factors of higher-order personalities [31,64].

The overall comparison between the group with internalizing disorders (diagnosis of depression or anxiety) and the control group showed statistically significant differences. The clinical group presented lower scores on the SEEKING and PLAY dimensions, i.e., scales that evaluate two positive affects, and higher scores on the ANGER and SADNESS dimensions, i.e., scales that measure negative affects. This first general analysis showed that subjects with psychopathological problems compared with controls had alterations in some of the neuroemotional systems identified by Panksepp [1]. These findings seem to highlight that, beyond the specific diagnostic picture, distress is linked to an impairment in primary emotional system functioning. In children with internalizing clinical symptoms, the desire for research and exploration towards the outside (SEEKING) and the pleasure of playing and engaging in playful activities with others (PLAY) were significantly reduced, while affective states linked to anger and frustration (ANGER) and feelings of sadness and pain due to separation from affective figures (SADNESS) were significantly increased.

Furthermore, the data obtained showed that children diagnosed with depression differed in neuroemotional system values compared with normotypic children in the control group. In particular, the group of depressed subjects had lower scores on the SEEKING, CARE, and PLAY dimensions and higher scores on ANGER and SADNESS. SEEKING, PLAY, and CARE represent the three primary emotional systems linked to positive affectivity since they correspond to enthusiasm for curiosity and the search for stimuli, the pleasure that accompanies playful activities, and the desire to take an interest in and take care of others [1]. Our results show that there is an impairment in these positive emotional aspects in depressed children, with a consequent tendency to experience affective states characterized by inactivity, disinvestment towards the outside, greater withdrawal from others, and less pleasure and involvement in playful activities. This latter aspect appears crucial in adaptive and developmental processes since play is considered a natural vehicle of expression for children from an evolutionary point of view. Toys are the words of children, and play is the language that allows them to express experiences [80,81]. Play is one of the most important ways in which children organize their experiences, express their emotions, and transform experiences in which the child is a passive subject into others in which the child is active [82]. Research has shown that the quality of play of depressed children is altered because they are less involved, the game is solitary–passive, and they do not show pleasure in sharing materials with others or communicating the playful activity they are carrying out [83,84]. Psychotherapies centered on the PLAY system use this important emotional system, common to mammals, to offer the possibility of experiencing a number of positive emotions, including joy, cheerfulness, excitement, interest, and pleasure, as well as a cathartic effect of negative emotions, such as anger and sadness [85,86,87].

Our finding of high ANGER and SADNESS values in depressed children is consistent with data on depressive characteristics in childhood, which highlight how children can manifest clinical symptoms with feelings of sadness or anger and irritability. In particular, high activation of the ANGER system is consistent with a series of studies that have reported greater aggression in samples with depressive disorders [88,89,90,91,92,93]. This finding seems to indicate the importance of anger as a signal of possible emotional distress, in which there could be a tendency to externalize discomfort since these feelings are so intense and unregulated that they are manifested through irritability and aggression [60]. As for the SADNESS dimension, which was higher in children diagnosed with depression, it must be emphasized how this primary emotional system is linked to pain and anxiety due to the loss of attachment bonds and separation from caregivers [1]. In particular, Panksepp argued that the pain caused by depressive symptoms is determined by initial hyperactivation of the PANIC system (called SADNESS in the ANPS test) following separation, which, if prolonged, leads to psychological despair [35]. In fact, the items on the scale contain statements that, in addition to investigating the states of suffering and sadness, also explore experiences of distress and anxiety due to separation from emotionally significant figures.

Research indicates that there is a considerable overlap between distress, anxiety, and depressive symptoms in childhood [94,95,96]. This overlap has led to the idea that there may be a temporal relationship between anxiety and depression so that anxiety disorders lead to concomitant depression [97,98,99,100]. In particular, some research has found a specific association between difficulty separating from parents and subsequent development of depression [101,102,103,104]. The high activation of the SADNESS scale in the clinical sample shows how difficulties separating from attachment figures and suffering can favor the development of depressive feelings, as observed by Panksepp [1] in animal studies, in which estrangement from significant carers caused strong painful affections related to the loss. This finding from affective neuroscience research seems to indicate the importance of (1) exploring the nature of attachment bonds in depressive manifestations of children since, as Bowlby [36] also pointed out, psychopathological problems can arise when there is no assurance of the physical and emotional closeness of caregivers, and (2) establishing treatment based on enhancing the quality of relationships between children and emotionally significant adults.

Finally, the results of this research found that subjects with anxiety disorder present, compared with the normative group, lower SEEKING and PLAY values and higher FEAR and SADNESS values. This would seem to indicate that anxious children show a decrease in the emotional systems dedicated to the search for stimuli in the environment, to interest, to the exploration of what surrounds them, and to the pleasure of playful activity, as if the state of anxiety was closely linked to a lower tendency to satisfy and seek stimuli and interests that generate feelings of being involved, effective, and gratified. Our results also highlighted an impairment in the SADNESS system, which indicates feelings of sadness and pain. The FEAR system was more activated in the group of anxious subjects than in the control group. This finding is consistent with the formulation proposed by Panksepp and colleagues [105], who highlighted the possible role of the FEAR system in anxious disorders, which could be characterized by hyperactivation of basic emotional processes related to fear. In fact, anxiety disorders are marked by excessive fear (and avoidance) of specific objects or situations in the absence of real danger. Neurobiological research on anxiety disorders has confirmed, through animal models, the involvement of fear circuits in animal models [106,107,108] and neuroimaging studies in humans [109,110,111,112].

The non-significant difference in the ANGER and CARE systems between anxious children and the control group seems to highlight that anxiety is not associated with imbalances in the expression of these neuroemotional systems. In fact, the state of worry and agitation of anxious subjects, as also found in other studies [113], would not seem to be linked to intense aggressive feelings, and would not seem to compromise the ability to be interested in others and support them. The CARE system concerns caring behaviors that can be compared with prosocial behaviors. Several studies [114,115,116] that have investigated the relationship between anxiety and prosocial behaviors in the development age have not found a significant effect. It is possible to hypothesize that anxious individuals may use prosocial behavior as a way to navigate social environments, unlike depressed individuals, who may lack the energy to engage prosocially with others, and unlike individuals with externalizing problems, who may be less concerned about others [115].

## 5. Conclusions

Our study provides a contribution to existing literature on ANPS by broadening the application of the tool to children and examining altered emotional systems in internalizing disorders, in particular in depressive and anxiety disorders in late childhood. 

First, the results of our study suggest that ANPS could be a diagnostic support to differentiate the different types of psychiatric disorders, or rather the different “emotional endophenotypes”. The ANPS could be used with other instruments within a battery, as it would allow for strengthening the diagnostic hypotheses through the identification of compromised neuroemotional systems, which, as we have seen in children with anxiety and depression, concern specific neuroemotional systems. Therefore, it could be interesting to extend the research to other psychopathological forms, such as externalizing problems, in order to use the ANPS as a clinical tool that facilitates the identification of emotional endophenotypes underlying psychiatric disorders.

Second, it emerged that the levels of neuroemotional systems of the normotypical group are significantly different from those of those suffering from a clinical problem. For this reason, the data of our study seem to encourage the investigation of the levels of neuroemotional systems in the general pediatric population in order to establish the range of normative values. Standardizing the instrument would allow us to have normative values and identify children who, despite difficulties in expressing their discomfort or being surrounded by adults who cannot understand their behavioral manifestations, are at risk of developing a psychopathological problem or present a neuroemotional alteration that indicates the presence of a clinical disorder.

Third, the results that emerged can provide indications to develop personalized interventions that can be more suitable for different clinical problems. Recognizing emotional endophenotypes can be useful to identify the most effective psychotherapeutic path, which, as highlighted by Panksepp [1], can completely replace pharmacological treatment in many cases, especially in childhood. In fact, the use of ANPS in the therapeutic field could help clinicians to identify the focus of the intervention. For example, our study has highlighted that, in both depressed and anxious children, some specific neuroemotional systems are compromised. Therefore, it could be useful to carry out a treatment, such as play therapy [87], that directly stimulates play skills (PLAY in both lower), allowing children to express and rebalance the activated emotions (FEAR and SADNESS for children with anxiety, ANGER and SADNESS for children with depression) and promote interest in themselves and the outside world (SEEKING for both clinical groups). In this regard, ANPS could also be used to conduct studies on the effectiveness of psychotherapy in the developmental age, allowing for integrating the evaluations of the symptomatic area and adaptation in life contexts, also the changes in the activation levels of neuroemotional systems.

The limitations of the present study are related to (**a**) the low Cronbach’s alpha value in some ANPS subscales, which could indicate the need to review the formulation of some items; (**b**) the small number of children belonging to the clinical group that limits the generalizability of the findings; (**c**) the fact that a self-report tool was used, which is subject to bias. Therefore, future studies should use a larger sample size and multiple investigation methods. Investigating the balance of dominant emotional systems could help clarify the neurobiological factors that play a key role in the onset of depression and anxiety. Over the last few decades, for example, several psychopharmacological studies have attempted to investigate the role of the noradrenergic and serotonergic systems in depression [117,118]. Furthermore, several genetic and epigenetic studies have tried to identify the role of the action of serotonergic transporter genes [119]. However, biogenic amines play a generic role in cellular excitability and impulse conduction and, therefore, do not appear to be specifically implicated in depressive disorders [35,120]. A more effective and targeted approach could therefore be to study neuropeptide and neurotransmitter system alterations relevant to basic neuroemotional systems, particularly possible alterations of the endogenous opioid system and the releasing hormone corticotropin (CRF) and the neurotransmitters glutamate and glycine [35]. The characterization, through ANPS, of the organization of dominant emotional systems in children with depressive and anxiety disorders could therefore be integrated with the study of the receptor polymorphism of genes that code for the opioid system (OPRM1), oxytocin (OXT), and vasopressin (AVP) and which, to date, seem to be involved in interindividual variability due to the manifestation of feelings of deep sadness, loneliness, anguish, and anger [121,122,123]. Although not addressed here, it should be noted that the environment plays a fundamental role in social and neuroaffective development. Therefore, investigating the interaction between genetics, basic affective organization, and the environment could be fundamental in the conceptual reformulation of depression and anxiety in childhood. This type of research could prove to be fundamental in understanding the neurobiological roots of the affective experiences typical of depressive and anxiety disorders and could offer new insights for basic research on psychopharmaceuticals. 

## Figures and Tables

**Table 1:** Correlations between the ANPS scales in the control group.

	SEEKING	FEAR	CARE	ANGER	PLAY	SADNESS
**SEEKING**	1.00	−0.09	0.13	−0.17	***0.27***	−0.18
**FEAR**	−0.09	1.00	0.16	0.20	−0.18	***0.65***
**CARE**	0.13	0.16	1.00	−0.09	***0.32***	0.20
**ANGER**	−0.17	0.20	−0.09	1.00	−0.18	***0.26***
**PLAY**	***0.27***	−0.18	***0.32***	−0.18	1.00	−0.23
**SADNESS**	−0.18	***0.65***	0.20	***0.26***	−0.23	1.00

**Table 2:** Correlations between the ANPS scales in the clinical group with internalizing disorders.

	SEEKING	FEAR	CARE	ANGER	PLAY	SADNESS
**SEEKING**	1.00	0.14	***0.28***	−0.07	***0.48***	0.08
**FEAR**	0.14	1.00	***0.31***	0.22	0.05	***0.42***
**CARE**	***0.28***	***0.31***	1.00	−0.09	***0.45***	0.20
**ANGER**	−0.07	0.22	−0.09	1.00	−0.20	***0.44***
**PLAY**	***0.48***	0.05	***0.45***	−0.20	1.00	−0.04
**SADNESS**	0.08	***0.42***	0.20	***0.44***	−0.04	1.00

## Data Availability

The raw data supporting the conclusions of this article will be made available by the authors on reasonable request.

## Abbreviations

The following abbreviations are used in this manuscript:

ANPS	Affective Neuroscience Personality Scales
